# Respiratory involvement and sleep-related disorders in CMT1A: case report and review of the literature

**DOI:** 10.3389/fneur.2023.1298473

**Published:** 2024-01-02

**Authors:** Sara Massucco, Cristina Schenone, Elena Faedo, Chiara Gemelli, Emilia Bellone, Lucio Marinelli, Davide Pareyson, Chiara Pisciotta, Tiziana Mongini, Angelo Schenone, Marina Grandis

**Affiliations:** ^1^Department of Neurosciences, Rehabilitation, Ophthalmology, Genetic and Maternal and Infantile Sciences (DINOGMI), University of Genoa, Genova, Italy; ^2^IRCCS Ospedale Policlinico San Martino, Genova, Italy; ^3^Rare Neurological Diseases Unit, Department of Clinical Neurosciences, Fondazione IRCCS Istituto Neurologico Carlo Besta, Milan, Italy; ^4^Neuromuscular Unit, Department of Neuroscience RLM, University of Torino, Torino, Italy

**Keywords:** CMT, Charcot–Marie–Tooth disease, CMT1A, sleep disorders, sleep apnea, respiratory disorders

## Abstract

Sleep-disordered breathing has been reported in Charcot–Marie–Tooth disease (CMT) type 1A in association with diaphragmatic weakness and sleep apnea syndrome, mainly of the obstructive type (OSA). Improvement has been observed not only in sleep quality but also in neuropathy symptoms in CMT1A patients with OSA following the initiation of continuous positive airway pressure. We report the cases of two siblings affected by CMT1A associated with hemidiaphragm relaxatio necessitating nocturnal non-invasive ventilation (NIV). Two twins, now 42 years old, with a family history of CMT1A, received a genetic diagnosis of CMT1A at the age of 16. Over the years, they developed a slowly worsening gait disorder and a decline in fine motor hand movements, currently presenting with moderate disability (CMTES:13). At the age of 40, they both started complaining of daytime sleepiness, orthopnea, and exertional dyspnea. They received a diagnosis of relaxatio of the right hemidiaphragm associated with impairment of nocturnal ventilation and they both have benefited from nocturnal NIV. Disorders of breathing during sleep may be underestimated in CMT1A since routine investigations of sleep quality are rarely performed. Our two clinical cases and a literature review suggest the importance of inquiring about symptoms of excessive daytime sleepiness and respiratory disturbances in individuals with CMT1A, even in the absence of severe neuropathy. In the presence of compatible symptoms, a pneumological assessment, along with an overnight polysomnogram and lung function tests, should be performed. Recognizing sleep-related symptoms is essential for providing accurate treatment and improving the quality of life for patients with CMT1A.

## 1 Introduction

Charcot–Marie–Tooth disease (CMT) refers to a heterogeneous group of hereditary progressive sensory-motor neuropathies with an overall prevalence of 9.37–20.1/100.000 (1). The classification of these neuropathies relies on nerve conduction velocity criteria, inheritance, and specific gene mutations (2). The prevalent forms typically follow an autosomal dominant pattern of inheritance. Among these forms are CMT1, characterized by demyelination and reduced nerve conduction velocity, accounting for most hereditary neuropathies, and CMT2, characterized by normal or minimally decreased nerve conduction velocity and axonal loss on pathological examination (3). The duplication of the peripheral myelin protein 22 (*PMP22*) gene results in CMT1A, which accounts for almost 40–50% of all diagnosed CMT cases (4–6).

Sleep and breathing disorders were first reported in subjects with severe CMT in association with phrenic neuropathy and diaphragmatic weakness (7, 8). Indeed, a high number of patients with CMT report daytime sleepiness and non-restorative sleep (9, 10).

Sleep disturbances may result from muscle cramps, paresthesia, neuropathic pain, sleep apnea, or restless legs syndrome (RLS). Sleep apnea, mainly of the obstructive type (OSA), has been reported to have a higher prevalence in patients with CMT (11–13). It can be diagnosed by polysomnography, and the apnea-hypopnea index (AHI) is typically used to measure the severity of OSA (14).

Moreover, vocal cord dysfunction and restrictive pulmonary impairment, which could result from phrenic nerve dysfunction or thoracic cage abnormalities, may also occur (15).

We herein describe the cases of two siblings affected by CMT1A associated with hemidiaphragm relaxatio necessitating nocturnal non-invasive ventilation (NIV).

## 2 Case presentation

Two 42-year-old twins were referred to our Neurological Outpatient Clinic with a two-year history of orthopnea and difficulty with sleep maintenance.

Their mother was affected by CMT1A. They had a history of foot deformities, specifically bilateral pes cavus and hammer toes, since childhood, as well as mild weakness of distal leg muscles that began in their second decade of life. Both siblings received a diagnosis of CMT1A at the age of 16 after the genetic detection of the duplication of the *PMP22* gene. They started using ankle-foot orthoses at the age of 35. Only one of the two brothers had kyphoscoliosis, for which he wore a corrective brace for two years starting when he was 10 years old. Neither had a history of alcohol abuse nor pulmonary diseases, although one of the brothers was a five-pack-year smoker. Their Body Mass Index (BMI) was 27.6 Kg/m^2^ in one patient and 27.8 Kg/m^2^ in the other one.

Neurological examination revealed severe hypotrophy and weakness of intrinsic hand muscles, as well as weakness in the muscles of the anterior compartment of the lower leg, accompanied by tingling paresthesias in the fingers and toes. Both siblings exhibited bilateral pes cavus, hammer toes, and a steppage gait with a CMT examination score (CMTES) of 13/28.

A nerve conduction study confirmed a significant slowing of conduction velocities, with a motor nerve conduction velocity of 20 m/s for the median nerve and 18 m/s for the peroneal nerve in both twins. The CMT neuropathy score (CMTNS) was 19/36 in both siblings.

Twenty-five years after the first symptoms, both patients developed orthopnea, requiring multiple pillows to sleep. Daytime somnolence was also reported.

The overnight polysomnography study confirmed the presence of mild obstructive sleep apneas and nocturnal hypoventilation with nighttime tonic desaturation. The apnea-hypopnea index (AHI) was 6.1 per hour in one sibling and 6.9 per hour in the other, while the mean peripheral oxygen saturation (SpO2) was 89 and 88%, respectively.

Pulmonary function tests suggested a restrictive defect. The first sibling had a forced expiratory volume in one second (FEV1) of 1.13 L (40% of normal), a forced vital capacity (FVC) of 1.30 L (39% of normal), a ratio of FEV1 to slow vital capacity (SVC) of 82 (97% of normal), a maximal expiratory pressure (MEP) of 96 cmH_2_O, and a maximal inspiratory pressure (MIP) of 42 cmH_2_O. The other brother also had an FEV1 of 0.90 L (31% of normal), an FVC of 1.20 L (35% of normal), a ratio of FEV1 to SVC of 73 (87% of normal), an MEP of 71 cmH_2_O, and an MIP of 28 cmH_2_O.

In both twins, the chest computed tomography (CT) showed a marked elevation of the right hemidiaphragm with minimal signs of ventilation impairment due to compression of the homolateral lung base and hypertrophy of the extrinsic respiratory muscles (scalene and pectoral muscles).

They were both initiated on non-invasive nocturnal ventilation with subsequent clinical benefit. After a few months, daytime sleepiness, assessed with the Epworth Sleepiness Scale, and morning headache disappeared. There was also an improvement in nocturnal oxygen saturation and arterial blood gas analysis (Table 1). Additionally, the AHI improved, with a residual AHI of less than one per hour in both siblings.

**Table 1:** Results of the exams before and after the initiation of nocturnal non-invasive ventilation.

Patient	Exam	Baseline	After the initiation of NIV
1	Cardio-Respiratory Monitoring	AHI 6.1/h, mean SpO_2_ 89%, CT90 33.8%	AHI < 1/h, mean SpO_2_ 92.8%, CT90 8.5%
	Arterial Blood Gas Analysis	pH 7.41, pO_2_ 75 mmHg, pCO_2_ 43 mmHg, HCO_3_^−^ 27.3 mmol/L	pH 7.45, pO_2_ 84 mmHg, pCO_2_ 40 mmHg, HCO_3_^−^ 27.6 mmol/L
2	Cardio-Respiratory Monitoring	AHI 6.9/h, mean SpO_2_ 88%, CT90 66.7%	AHI < 1/h, mean SpO_2_ 96%, CT90 0%
	Arterial Blood Gas Analysis	pH 7.43, pO_2_ 69 mmHg, pCO_2_ 40 mmHg, HCO_3_^−^ 26.4 mmol/L	pH 7.44, pO_2_ 74 mmHg, pCO_2_ 39 mmHg, HCO_3_^−^ 26.5 mmol/L

NIV = non-invasive ventilation; AHI = apnea-hypopnea index; SpO2 = mean nocturnal oxygen saturation; CT90 = cumulative percentage of time with SpO2 less than 90%.

## 3 Review of the literature and discussion

Sleep and breathing disorders in patients with CMT are likely underreported, as they were initially described only in severe forms of neuropathy, and pulmonary function tests are not routinely conducted in CMT patients. Additionally, most patients described so far were older.

We herein report the case of two 40-year-old twins with a moderate form of CMT1A who exhibited an elevated hemidiaphragm and respiratory disturbances at a young age.

A significant number of individuals diagnosed with CMT experience respiratory muscle weakness, which is associated with restrictive physiology (15). Pulmonary function tests sometimes reveal abnormalities even in asymptomatic CMT1 patients. Notably, many authors have described a high prevalence of alterations in FVC, MIP, and MEP (16–18).

A study by Junior and colleagues investigated the association of neurological status and respiratory parameters in wakefulness and sleep with the physiology and morphology of phrenic nerves in 16 patients with CMT1A (19). The study revealed a significant correlation between CMTNS and AHI, CMTNS and MEP, as well as CMTNS and MIP. The reduction in Compound Muscle Action Potentials (CMAPs) did not correlate with MIP or MEP in this series, underscoring the importance of a comprehensive evaluation of respiratory function in CMT1 patients. Ultrasonography detected an enlargement of phrenic nerves, and a significant negative correlation between the cross-sectional area and the CMAPs of the phrenic nerves was observed (19). Restrictive respiratory dysfunction and a slight diaphragm elevation were identified in only one patient with very severe neuropathy (19). The authors hypothesized that the high prevalence of respiratory weakness in patients with CMT1A may be attributed to the axonal degeneration of nerves innervating respiratory muscles.

Multiple case reports suggest a potential link between CMT and impairment of the diaphragm or phrenic nerve (7, 8, 20, 21), and both neurophysiological and pathological evidence of phrenic nerve involvement exists (16, 22, 23). Furthermore, individuals with CMT may exhibit spinal deformities such as scoliosis or kyphoscoliosis, which could be associated with a decrease in lung volume independent of any phenic nerve dysfunction (15). Neuropathic spinal arthropathy has been proposed as a potential mechanism through which CMT may contribute to the development of spinal deformities (24).

Studies investigating excessive daytime sleepiness in individuals with myotonic dystrophy have at times employed patients with CMT as the control group, assuming the absence of central nervous system involvement in CMT. One study reported a high frequency of insomnia because of cramps, while another found an elevated rate of daytime hypersomnolence in CMT patients (25, 26). Boentert and colleagues described higher fatigue, a greater prevalence of daytime sleepiness, and poorer sleep quality compared to controls in a large cohort of patients with CMT (9). Notably, the prevalence of restless legs syndrome (RLS) was 18.1% in CMT patients (versus 5.6% in controls), with higher frequency and severity in females than males (9). RLS was initially described only in patients with the axonal forms of CMT (or CMT2). In a study with 44 participants by Gemignani et al., 37% of CMT2 patients and 0% of CMT1 patients had RLS (27). Other studies report a prevalence of 16.1% in CMT2 and 10% in CMT1 (28), or even a higher prevalence of RLS in CMT1 than in CMT2 (9). Either way, axonal damage is believed to predispose individuals to RLS, and female CMT patients are more often affected than males. A recent study by Bellofatto and colleagues confirmed the frequent presence of sleep disorders and daytime sleepiness in patients with CMT (29). A significant difference in the Pittsburgh Sleep Quality Index (PSQI) was observed between subjects with CMT and controls. Patients with PSQI >5 also exhibited higher CMTES and greater use of antidepressant and anxiolytic medications (29). The same authors also assessed the prevalence of fatigue in a large series of CMT patients recruited through the Italian CMT Registry (30). Thirty-six percent of CMT patients had abnormal fatigue based on the Modified Fatigue Impact Scale (MFIS), and patients with fatigue exhibited higher CMTES, an increased need for foot orthoses and walking support, greater hand disability, and positive sensory symptoms. Patients with abnormal fatigue also showed a higher frequency of anxiety/depression, daytime sleepiness, obesity, and use of antidepressants/anxiolytics or anti-inflammatory/analgesic medications (30).

Dematteis et al. and Dziewas et al. reported an increased prevalence of sleep apnea, mainly but not exclusively of the obstructive type, in patients with CMT1A (11, 12). The severity of obstructive sleep apnea (OSA), as assessed by the apnea-hypopnea-index (AHI), correlated with disability due to CMT (12). Additionally, Boentert and colleagues studied 61 subjects with CMT1, confirming that patients with CMT1 are more often affected by OSA than control subjects matched for age, sex, and other risk factors of OSA, such as overweight and obesity (13). Neurological disability, as assessed by the Functional Disability Scale (FDS) score, emerged as an independent predictor of OSA in CMT1A patients (13). They hypothesized that sleep-disordered breathing may be linked to phrenic neuropathy and diaphragmatic weakness only in severely affected CMT1A patients, while different causes need to be studied in milder cases. Even though this assumption is acceptable, our patients showed evidence of diaphragmatic dysfunction despite having only a moderate neurological disability. Furthermore, pharyngeal and phrenic nerves are affected in CMT1A, but OSA and respiratory disorders do not occur in all CMT1A patients. Unfortunately, the phrenic nerve conduction study was not performed in our patients, and this certainly represents a limitation of the study.

The mechanism behind the increased risk of OSA in patients with CMT is not entirely understood, but many authors have proposed pharyngeal neuropathy due to CMT and increased upper airway compliance as the main risk factors (12, 13). In CMT patients, neuropathy may impair the stabilizing function of the pharyngeal muscles and the local mechanisms that prevent the collapse of upper airways during inspiration, leading to increased upper airway collapsibility (11). Severe CMT neuropathy could lead to OSA even in the absence of other risk factors such as old age, obesity, or facial dysmorphism (11). Additionally, OSA itself represents a potential aggravating factor for neuropathy, and continuous positive airway pressure (CPAP) is the first-line treatment (11). Since OSA in CMT is likely caused by denervation rather than mechanical issues, lower CPAP levels may be sufficient to reopen closed airways (31), making the treatment of OSA due to CMT potentially easier than that of OSA from other causes. However, considering the diaphragmatic dysfunction necessitating noninvasive ventilation, our patients were initiated on nocturnal bilevel positive airway pressure instead of CPAP, and their symptoms significantly improved. Indeed, for patients with both sleep apnea and restrictive pulmonary disorder, bilevel-positive airway pressure is likely more appropriate than CPAP (15).

Repetitive nighttime drops in oxygen levels can cause frequent awakenings and sleep deprivation. Without early recognition, severe obstructive sleep apnea-hypopnea significantly raises the risk of cardiovascular events. Using CPAP has been shown to mitigate this risk (32). Some case reports even suggest that treating OSA in CMT patients can alleviate neuropathy symptoms (33). There is speculation about OSA causing peripheral nerve damage (34, 35). Intermittent hypoxemia may cause partially reversible sensory nerve damage through functional changes (35), and CMT1A nerves might be particularly sensitive to hypoxemia (33). Notably, a woman with CMT1A experienced relief from numbness and cramps after starting CPAP for OSA (33).

The presence of sleep apnea in a patient with CMT may also heighten the susceptibility to perioperative airway complications, underscoring the importance of assessing symptoms related to sleep apnea before undergoing surgery (15).

Respiratory issues could heighten the risk of complications in the case of respiratory infections, including Severe Acute Respiratory Syndrome Coronavirus 2 (SARS-CoV-2). The onset of respiratory symptoms was subtle in our patients, and a slow worsening occurred, so they did not undergo a nasopharyngeal swab to check for possible SARS-CoV-2 infection. Considering the evidence supporting vaccination in patients with polyneuropathy (36), both patients received multiple doses of the anti-SARS-CoV-2 mRNA-based vaccine. They reported no SARS-CoV-2 infection, yet the potential role of an unrecognized infection at the onset of respiratory symptoms cannot be ruled out.

Sleep-related disorders significantly impair the quality of life in individuals with CMT (12, 13). However, since routine investigations of sleep quality are rarely performed, disorders of breathing during sleep in CMT patients are likely underestimated. Our two clinical cases, along with the available literature, suggest that individuals with CMT should be asked about symptoms of excessive daytime sleepiness and respiratory disturbances, even in the absence of severe neuropathy or at a young age. If compatible symptoms are present, an overnight polysomnogram and lung function tests should be conducted. Respiratory issues in CMT are more common than previously thought and are likely underestimated. Still, they can lead to a deterioration in quality of life, increase cardiovascular risk, and pose perioperative complications. It would thus be important, even for patients with mild forms of CMT, to undergo a thorough pulmonary evaluation.

Even if a causative treatment for CMT is not yet available, the prompt recognition of sleep-related disorders may help improve patients’ quality of life and prevent cardiopulmonary complications.

## Ethics statement

Written informed consent was obtained from the individual(s) for the publication of any potentially identifiable images or data included in this article.

## Author contributions

SM: Conceptualization, Investigation, Writing – original draft, Writing – review & editing. CS: Conceptualization, Writing – original draft. EF: Investigation, Writing – original draft. CG: Conceptualization, Investigation, Writing – original draft. EB: Data curation, Supervision, Writing – review & editing. LM: Investigation, Writing – review & editing. DP: Investigation, Writing – review & editing. CP: Investigation, Writing – review & editing. TM: Investigation, Writing – review & editing. AS: Conceptualization, Supervision, Writing – review & editing. MG: Conceptualization, Supervision, Writing – review & editing.
